# *ACTN3*, Morbidity, and Healthy Aging

**DOI:** 10.3389/fgene.2018.00015

**Published:** 2018-01-24

**Authors:** Craig Pickering, John Kiely

**Affiliations:** ^1^Institute of Coaching and Performance, School of Sport and Wellbeing, University of Central Lancashire, Preston, United Kingdom; ^2^Exercise and Nutritional Genomics Research Centre, DNAFit Ltd., London, United Kingdom

**Keywords:** *ACTN3*, genetics, aging, sarcopenia, bone mineral density, personalized

## Abstract

As human longevity increases, recent research has focused on the maintenance of optimal health during old age. One such area of focus is that of muscle function in the elderly, with a loss of muscle mass increasing the risk of negative outcomes such as sarcopenia and a decrease in bone mineral density. In this mini-review, we focus on the impact of a single nucleotide polymorphism in *ACTN3*, shown to impact muscle phenotype in elite athletes, on loss of muscle function, maintenance of bone mineral density, and metabolic disorder risk in an elderly population. From the surveyed research, this polymorphism has a clear and demonstrable impact on muscle phenotype and bone mineral density in this population, and acts as a potential modulator for metabolic disorders. As such, knowledge of an individual’s *ACTN3* genotype may better inform the management of risk factors in the elderly, as well as driving innovations in exercise program design. Subsequently, such insights may contribute to the prolonged maintenance of health and function long into old age.

## Introduction

There is a frequently quoted axiom, often attributed to Benjamin Franklin, suggesting that “nothing is certain but death and taxes.” Whilst recent scandals suggest that, for some, taxes may be optional, death remains a universal certainty. Fortunately, life expectancy has increased dramatically over a very short time-frame. Within the United Kingdom, for example, the expected lifespan has roughly doubled over the past 150 years, such that a child born today can expect to live until 80 years of age (Majeed, 2013). Whilst reductions in infant mortality undoubtedly play a role, they only provide a partial explanation. This substantial leap in life expectancy is attributable to multiple – medical, societal, cultural, economic, and public health – factors. As a consequence, the number of people surviving into old age is rising, a trend which is expected to continue (He et al., 2016).

This trend has piqued interest in healthy aging, particularly as longer lifespans don’t always correlate with sustained wellbeing (Christensen et al., 2009; Kuh et al., 2014). As health is multifactorial, the research in this field has a wide scope, including disease avoidance and the maintenance of physical function into old age (Christensen et al., 2009; Kuh et al., 2014). Focusing on the latter, a number of physical performance measures are associated with healthy aging, including grip strength, standing balance, and walking speed, with lower scores in these tests typically associated with increased all-cause mortality (Rantanen, 2003; Cooper et al., 2010; Studenski et al., 2011). As such, along with the absence of disease states such as type-II diabetes, muscle strength is an important component of healthy aging.

A second population to which muscle strength is important are elite athletes (Maughan et al., 1984; Häkkinen and Keskinen, 1989). With both muscle strength and elite athlete status being heritable traits (De Moor et al., 2007; Silventoinen et al., 2008), over the last 20 years there has been an increased focus on identifying the specific genes and single nucleotide polymorphisms (SNPs) impacting the inter-individual variation evident in athletic performance (Hughes et al., 2011; Timmons, 2011). At present, over 100 SNPs associated with elite athlete status (Ahmetov and Fedotovskaya, 2015) and the exercise training response (Bray et al., 2009) have been identified. One such SNP with a well-established influence on muscle phenotype is rs1815739, a C-to-T base substitution in *ACTN3* (Yang et al., 2003; Ma et al., 2013). This SNP results in the transformation of an arginine base (R) to a premature stop codon (X), with X allele homozygotes deficient in the α-actinin-3 protein (North et al., 1999). The main function of α-actinin-3 appears to be as a structural protein, forming part of the Z-line of the muscle fiber, which acts to anchor the actin filaments within the sarcomere (Yang et al., 2009). This protein is expressed exclusively in type-II muscle fibers, and as a result, XX genotypes tend to have a lower percentage of these fibers (Vincent et al., 2007). As such, the XX genotype is significantly under-represented in elite speed, power, and strength athletes (Yang et al., 2003; Roth et al., 2008), although these results are not unequivocal (Scott et al., 2010; Sessa et al., 2011).

Both strength and muscle mass are protective against all-cause mortality in the elderly (Li et al., 2017). As *ACTN3* genotype can modify muscle phenotypes, this narrative mini-review will explore the relationship between this common polymorphism in *ACTN3* and healthful aging, with a particular focus on muscle. Such exploration provides a basis for an enhanced understanding of individualized risk factors for the morbidities associated with the aging muscle, and may soon guide the customization of prophylactic exercise interventions such as resistance training.

## *ACTN3*, Muscle Mass, and Healthy Aging

Sarcopenia is the loss of skeletal muscle mass and function associated with increased age (Rosenberg, 1997; Cruz-Jentoft et al., 2010). This process begins relatively early in life, with reported onset at age 25 (Lexell et al., 1988), a 10% loss in peak lean mass at age 40, and 40% loss at age 70 (Porter et al., 1995). This loss of muscle mass and strength can be troubling for a variety of reasons, such as a reduction in overall function (Janssen et al., 2002; Rantanen, 2003) and an increase in fall risk (Wickham et al., 1989). In knock-out (KO) mouse studies, those without *ACTN3* have a greater muscle mass loss with aging (Seto et al., 2011); are these results mirrored in humans?

A number of studies have examined the impact of *ACTN3* on muscle strength and function in an elderly population. Delmonico et al. (2008) undertook an observational study of over 3000 well-functioning elderly subjects over a 5-year period. In males, increases in 400 m walk time were significantly greater in XX homozygotes than RR genotypes, with a non-significant difference between XX homozygotes and RX genotypes (*p* = 0.075). In females, RR genotypes had approximately a 35% lower risk of persistent lower extremity limitation (defined as difficultly walking 400 m or climbing 10 steps without resting) than XX genotypes. Interestingly, there were no significant differences between genotypes with regards to other muscle and performance phenotypes. Kikuchi et al. (2015) reported a similar loss of function in elderly Japanese subjects, with a significantly poorer chair stand test score in XX genotypes compared to RR and RX genotypes. Judson et al. (2011) examined *ACTN3* genotype interaction on fall risk in over 4000 elderly Caucasian females. Here, subjects with at least one X allele had a significantly increased risk of falling than R allele carriers; this was true at both baseline and at multiple follow-up points. These results were mirrored by Frattini et al. (2016), who reported that falls were more prevalent in XX genotypes than R allele carriers. Walsh et al. (2008) reported that, in females, the XX genotype was associated with significantly lower total-body and lower-limb fat free mass (FFM). In addition, these female subjects had lower peak torque values compared to R allele carriers. There were no genotype effects in male subjects. Similar lower values for muscle mass in elderly female XX homozygotes were reported by Zempo et al. (2010), with mean thigh cross-sectional area 4.5 cm^2^ lower in XX vs. R allele carriers (*p* < 0.05). Finally, Cho et al. (2017) reported a significantly higher sarcopenia risk in XX genotypes than RR genotypes in a cohort of elderly Koreans. However, other studies have found no effect of this polymorphism on muscle phenotype and function in the elderly (San Juan et al., 2006; Bustamante-Ara et al., 2010; McCauley et al., 2010), and one study (Lima et al., 2011) reported significantly greater FFM values in X allele carriers.

The general consensus from these studies is that *ACTN3* genotype exhibits a potentially modifying effect on muscle mass, maintenance of muscle function, and sarcopenia risk in elderly subjects, with the R allele associated with greater maintenance of strength and function, and sarcopenia protection. From a muscle phenotype perspective, an association between *ACTN3* genotype and sarcopenia seems logical; specific type-II muscle fiber atrophy is a hallmark of sarcopenia (Lexell et al., 1988; Fielding et al., 2011), and, in athletic populations at least, the R allele is associated with an increase in type-II muscle fibers (Vincent et al., 2007). This ability to more effectively maintain fast-twitch fiber size and mass with age is perhaps the mechanism by which *ACTN3* genotype modifies the age-related loss in muscle function, and concurrent increased fall and sarcopenia risk.

Given that resistance training is an important tool in sarcopenia prevention and treatment (Roth et al., 2000), and that *ACTN3* genotype may modify resistance training adaptations (Kikuchi and Nakazato, 2015), it is important to explore whether such a relationship exists in an elderly population. In elderly Caucasian females undertaking a 12-week resistance training program, Pereira et al. (2013) reported that *ACTN3* RR genotypes exhibited greater leg extension and bench press one-repetition maximum (1RM) improvements than XX genotypes. Delmonico et al. (2007) put elderly subjects through a 10-week unilateral knee extensor strength training program. In the male sub-group, absolute peak power increased to a greater extent in RR homozygotes compared to XX homozygotes, although this difference was not significant (*p* = 0.07). In females, relative peak power change was greater in the RR group compared to the XX group. As far as we are aware, these are the only two studies to examine the impact of *ACTN3* on resistance training response in an elderly cohort, with the consensus being that the R allele, and specifically the RR genotype, is associated with enhanced strength and power improvements. Based on these findings, it appears that elderly R allele carriers are more responsive to resistance training.

## *ACTN3* Genotype and Bone Mineral Density With Aging

Alongside age-related loss of muscle mass and function, a further risk factor is the loss of bone mineral density (BMD) and its related disease state, osteoporosis, with a well-established association between lower BMD scores and increased all-cause mortality (Browner et al., 1991; Johansson et al., 1998), stroke death (Browner et al., 1991), and fracture risk (Marshall et al., 1996). A small number of studies have examined the interaction between *ACTN3* genotype and BMD loss in elderly populations. Min et al. (2016), for example, reported a significant difference in BMD at both the spine and pelvis between genotypes, with XX and RX genotypes having lower scores than RR genotypes. Cho et al. (2017) reported similar findings, although the lower BMD in XX genotypes wasn’t significant after covariate correction (*p* = 0.075). Yang et al. (2011) found that, in postmenopausal women, *ACTN3* genotype was significantly associated with BMD, with XX genotypes having the lowest scores. Accordingly, overall it appears that the *ACTN3* R allele is somewhat protective against age-related BMD loss.

As discussed, *ACTN3* genotype is likely associated with muscle function in the elderly. This may be the driving force between genotype differences in BMD, with individuals possessing greater muscle function able to be more active day-to-day. Such individuals are subsequently more likely to experience regular skeletal loading, thereby promoting structural maintenance, and diminishing BMD loss over time. Indeed, grip strength is positively correlated with BMD (Iida et al., 2012), as is increased muscle mass (Visser et al., 1998), indicating that perhaps the increased muscle mass and strength associated with the R allele is protective in this manner. However, using KO mice, Yang et al. (2011) reported a lower BMD in mice deficient in α-actinin-3. They reported evidence that a-actinin-3 is expressed in bone tissue and involved in osteogenesis, with KO mice having a reduced osteoblast and increased osteoclast activity. Perhaps both mechanisms play a role in the relationship between *ACTN3* and BMD, with further research required to understand the relative contributions of each.

## *ACTN3* Genotype and Metabolic Health With Aging

Alongside muscle and BMD loss, aging populations also have to contend with an increased prevalence of a number of metabolic issues, including insulin resistance and type-II diabetes (Gunasekaran and Gannon, 2011; Suastika et al., 2012). These disease states are associated with a reduced mortality (Panzram, 1987), as well as an increased risk of further health issues (Williams et al., 2002) and cognitive decline (Strachan et al., 1997). Given that higher levels of muscle mass are associated with better insulin sensitivity (Srikanthan and Karlamangla, 2011), and that *ACTN3* genotype can modify muscle cross sectional area and fiber type, there is the potential that *ACTN3* genotype may impact type-II diabetes risk, either directly or indirectly. There is a paucity of research in this area; however, Riedl et al. (2015) reported that the prevalence of XX genotypes was greater in type-II diabetes patients than controls, indicating that it may increase risk, although there were no differences between genotypes in terms of metabolic control or obesity. Research on *ACTN3* KO mice indicates that deficiency of Actn3, characterized by the XX genotype, does alter skeletal muscle metabolism (MacArthur et al., 2007), potentially by increasing fatty acid oxidation and glycogen storage.

As of yet, any relationship between this SNP and type-II diabetes requires further elucidation. The tentative findings of Riedl et al. (2015) are further complicated by research on the relationship between *ACTN3* and extreme longevity. In a cohort of Spanish centenarians, the XX genotype frequency was the highest reported in non-athletic Caucasians (24%), although there were no significant differences between X allele frequency in centenarians and controls (Fiuza-Luces et al., 2011). The authors concluded that this preliminary data suggests a potential survival advantage of the XX genotype. Similar complex results were found in a cohort of Japanese centenarians. Whilst there were no significant differences in genotype distribution between centenarians and controls, the frequency of the XX genotype in supercentenarians (over 110 years) was the highest seen in a non-American population, at 33% (Fuku et al., 2016). Indeed, whilst it appears that the evidence suggests that the R allele may confer a longevity advantage, likely mediated through its impact on muscle function, bone health, and metabolic wellbeing as discussed in this review, the lack of increased RR genotype frequencies seen in centenarians (Fiuza-Luces et al., 2011; Fuku et al., 2016) does not support this. Such a finding is mirrored in the longevity of elite athletes, with elite endurance athletes tending to live for longer than power athletes (Sarna et al., 1993; Teramoto and Bungum, 2010; Clarke et al., 2015). As the R allele is more prevalent in elite power athletes than elite endurance athletes (Yang et al., 2003), this again appears to suggest a paradox. The mechanisms underpinning the longevity advantage of elite endurance athletes is currently unclear, although there is the potential that the enhanced cardiorespiratory fitness exhibited by elite endurance athletes offers greater longevity than the improved muscle strength and function expected in former elite power athletes (Wisløff et al., 2005). This is particularly pertinent given evidence of more efficient aerobic metabolism in XX homozygotes (North, 2008). Alternatively, the X allele could confer some as of yet unclear survival benefit; if this is the case, then there is the possibility that RX heterozygotes may have the greatest longevity benefit, by enjoying the benefits associated with each allele. Such an explanation would provide a potential mechanism explaining the lack of expected increases in RR genotypes in centenarian populations.

Nevertheless, given that loss of muscle mass increases risk of insulin resistance, a precursor to type-II diabetes (Srikanthan and Karlamangla, 2011), and that type-II diabetes itself increases the risk of sarcopenia (Park et al., 2009; Kim et al., 2010), it appears that *ACTN3* genotype may modify type-II diabetes risk in the elderly. Again, it would be expected that the R allele, which is associated with increased muscle mass and performance, would be protective against age-related metabolic decline. Further research in this field should attempt to uncover such a relationship, should one exist.

In addition, *ACTN3* may alter health through other metabolic disturbances. In mouse models, there is evidence that the XX genotype may be protective against obesity (Houweling et al., 2017), although as of yet this association has not been replicated in humans (Moran et al., 2007; Houweling et al., 2017), with Deschamps et al. (2015) reporting increased obesity in XX genotypes. Similarly, there is evidence in younger populations that this polymorphism may impact other health markers, such as blood pressure (Deschamps et al., 2015) and high-density lipoprotein cholesterol (Nirengi et al., 2016); in both cases, the X allele was beneficial, although it’s not clear if this clinically meaningful, with further replication required.

## Is This Trifecta Caused by *ACTN3*’s Influence on Muscle?

In this paper, we have examined the potential influence of *ACTN3* on three conditions associated with poorer outcomes with aging; sarcopenia and the resulting loss of muscle function, a loss of BMD, and a potential increase in metabolic disturbances, such as insulin resistance. These conditions likely have some degree of inter-relation; a loss of muscle function is likely associated with a lack of movement, which in turn reduces bone loading and turnover, leading to a loss of BMD (Vincent and Braith, 2002; Korpelainen et al., 2006). This loss of movement capacity could further cause a behaviorally mediated loss of type-II muscle fibers, further reducing muscle strength and function. Again, this loss of function might change habitual movement behaviors, thereby subsequently altering the metabolic profile of the individual and increasing the likelihood of some negative metabolic changes.

Accordingly, it seems feasible to speculate that the impact of *ACTN3* on these three risk-factors occurs either due to its directly modifying effect on skeletal muscle, or through separate mechanisms for all three. This raises the question of whether elderly X allele carriers have lower BMD because they have less muscle mass and function, or if there is mechanism through which *ACTN3* influences bone turnover and mineral content. As detailed in Section “*ACTN3* Genotype and Bone Mineral Density with Aging,” there are tentative results that *ACTN3* genotype influences both of these considerations, although whether its influence is greater on one than the other is currently unclear. As the results regarding *ACTN3* and insulin resistance are under-explored (Riedl et al., 2015), this leg of the trifecta is the most unknown; whilst there is a mechanism underpinning muscle mass and insulin resistance (Srikanthan and Karlamangla, 2011), and *ACTN3* does modify muscle mass and type in athletic cohorts (Vincent et al., 2007), it isn’t clear whether this holds true in the elderly.

If, as seems likely, the potentially modifying impact of *ACTN3* genotype on these three morbidities occurs primarily, although not exclusively, through its role in regulating muscle fiber type and strength, then this further underscores the need for elderly adults to undertake resistance training in order to maintain their health and function as they age. Whilst there is a clear protective effect of resistance training on the reduction of sarcopenia (Johnston et al., 2008), enhancing BMD (Rhodes et al., 2000), and reducing risk of insulin resistance and type-II diabetes (Dunstan et al., 2002) in the elderly, the insights outlined here do suggest some additional questions. Do those with the XX genotype, who would be expected to exhibit small improvements with resistance training, need to increase their training frequency and/or intensity (as suggested with regards to aerobic endurance training by Montero and Lundby, 2017), or should they undertake lower-load, higher-volume resistance training, as suggested by Kikuchi and Nakazato (2015) and supported by Jones et al. (2016)? Do other SNPs, such as those found in *ACE* (Pescatello et al., 2006) or *AGT* (Aleksandra et al., 2016), influence the resistance training response in the elderly, and to what extent? There is also the possibility that *ACTN3* genotype may interact with other SNPs to modify the aging process in individuals. This has perhaps been most well studied in regard to *ACE* I/D, a SNP in the gene encoding for angiotensin-converting enzyme. Here, the results are equivocal, with some studies finding no effect of the *ACE* I/D polymorphism on muscle phenotype (McCauley et al., 2010; Garatachea et al., 2012), and others reporting that it modified the response to resistance training (Pereira et al., 2013), both on its own and in combination with *ACTN3.* Like *ACTN3, ACE* may also impact longevity through a variety of different pathways, including metabolic disease risk (Kajantie et al., 2004), blood pressure control (Yoshida et al., 2000; Santana et al., 2011), and Alzheimer’s disease risk (Narain et al., 2000). Further work exploring the impact of resistance training on the elderly should perhaps take into consideration differences in genotype, either for single or multiple SNPs, to inform the design of more efficient and effective personalized exercise guidelines targeting positive outcomes for this population.

## Conclusion

**FIGURE 1:**
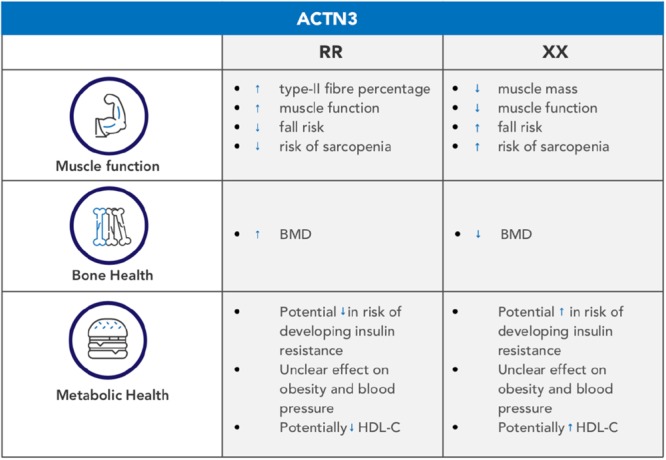
An overview of the main associations between *ACTN3* and healthy aging.

*ACTN3* has a demonstrable, clear and robust effect on muscle phenotypes in young, athletic populations (MacArthur and North, 2007; Vincent et al., 2007). Based on the research cited in this review, it appears to have a modifying effect on muscle strength, size and function in the elderly (Delmonico et al., 2008; Walsh et al., 2008; Frattini et al., 2016), as summarized in **Figure 1**. In particular, the R allele of *ACTN3* tends to be associated with better maintenance of muscle mass, strength and function (Delmonico et al., 2008), a greater adaptive response to training (Pereira et al., 2013), and is protective against the development of sarcopenia (Cho et al., 2017). There also appears to be a (less robust) relationship between *ACTN3* genotype and BMD in the elderly, with the R allele again being protective (Min et al., 2016; Cho et al., 2017). It is not clear whether this is due to *ACTN3* directly influencing bone metabolism, or whether the increased muscle mass and function of R allele carriers leads to greater bone loading, and therefore BMD maintenance. Similarly, there is an unclear relationship between *ACTN3* genotype and metabolic health; one study (Riedl et al., 2015) indicates that the XX genotype is present with an increased frequency in type-II diabetes patients, but clearly further research is required to better understand this relationship. Overall, whilst this indicates that the R allele should be associated with increased health and function in the elderly, the picture is made more complex by research on centenarians (Fiuza-Luces et al., 2011; Fuku et al., 2016); in this case, the XX genotype is potentially more frequent in those over 100 years of age, although such a relationship is not statistically significant. If further research does support the early evidence that the *ACTN3* R allele is associated with a decrease in frailty risk factors, then knowledge of *ACTN3* genotype may better inform patients and medical practitioners as to each individuals’ risk factors. Such information could consequently inform personalized management strategies for the aging individual.

## Author Contributions

CP conceived the idea for the manuscript and authored the first draft, and edited later drafts. JK provided substantial feedback on this draft, aiding in further analysis of the existing literature, and rewrote large sections of the manuscript. Both authors give approval for this version of the manuscript to be published, and agree to be accountable for the content contained within.

## Conflict of Interest Statement

CP is an employee of DNAFit Ltd., a genetic testing company. He received no payment for the production of this article, which was completed as part of his Professional Doctorate studies at the University of Central Lancashire. The other author declares that the research was conducted in the absence of any commercial or financial relationships that could be construed as a potential conflict of interest.
